# Supplementary material for: Tissue Characteristics in Endodontic Regeneration: A Systematic Review
Source: Int J Mol Sci. 2022 Sep 11;23(18):10534. doi: 10.3390/ijms231810534 (PMC9504778; doi:10.3390/ijms231810534)
Supplement: Supplementary file 1 [file ijms-23-10534-s001.zip › ijms-1874595-supplementary.pdf]

International Journal of
Molecular Sciences

MDPI

Annex Table

	Clinical context	Follow-up	Materials or REP procedure	Assessment	Results
Porcine model					
**Jang et al. 2020 (34)**	REP<br>Healthy immature premolar teeth	1-3 months	MTA<br>Extrinsic synthetic gelatin-based matrix (Floseal, Baxter, CA, USA)<br>Extrinsic synthetic fibrin-based matrix (Greenplast-Q, Green-Cross, Seoul, Korea)	**2D Radiography<br>Histology**	**<u>Histological & radiographic examination</u>**<br>1) pulp-like tissues and deposition of newly formed tertiary dentin with apex maturation<br>2) No intraradicular mineralized tissue deposition microvasculature and odontoblastic layers aligned on the root dentin<br>3) Infiltration of inflammatory cells and the resorptive lesions with the presence of inflammatory cells were observed in the root canal system
**Guo et al. 2021 (33)**	Bioengineered incisor tooth implantation	6 months	natural DTM combined with autologous stem cell aggregates	**Histology<br>Immunohisto-chemistry<br>SEM**	**<u>Histological observation</u>**<br>1) Bioengineered implanted teeth regenerated pulp tissue<br>- Odontoblastic layer<br>- Blood vessels on coronary third<br>2) Periodontal tissue regeneration + cementum cells, Sharpey's periodontal fibers and blood vessels on apical third

**Dog model**					
**Siddiqui et al. 2021 (35)**	REP infected mature incisors & canine	7,14, 28 days	SLan angiogenic target peptide. Vs SLed dentinogenic control peptide	**Histology Immunohistochemistry**	<u>**Histological observation**</u><br>1) Sled failed revascularization<br>2) Slan Successful revitalization<br>- Poor disorganized tissue and soft tissue in the canal<br>- Presence of vessels<br>- Nerve bundle<br>- Odontoblast-like layer on intracanal dentin<br>- Calcification or disorganized hard tissue<br>- Significant cellular infiltration

**Kim et al. 2021 (36)**	REP<br>Healthy mature premolar teeth	12 weeks	Collagen membrane vs amnion-chorion membrane + calcium silicate–based cement	**Histology**	**<u>Histological observation</u>**<br><u>Collagen membrane</u><br>1) Mesial root showing regenerated fibrous tissue and mineralized tissue<br>2) Island of regenerated mineralized tissue partly attached to the native dentin<br>3) Sharpey's fiber-like fibrous tissue attachment to the regenerated mineralized tissue and the lining of cells that are not odontoblast-like.<br>4) Newly regenerated mineralized tissue onto the native dentin.<br>5) Root apex showing apical closure with apical ramifications and no periapical inflammation.<br><u>ACM</u><br>1) Odontoblast-like cells were identified in 75% of the roots<br>2) Fibrous connective tissue with perfused blood vessels<br>3) Mesial root showing regenerated fibrous connective tissue without periapical inflammation.<br>4) Root apex showing apical closure with apical ramifications and no periapical inflammation.

**Abbas et al. 2020 (37)**	REP<br>Infected immature premolar teeth	1 week, 1 month, 3 months	Chitosan loaded<br>- demineralized bone matrix<br>- dexamethazone corticosteroid<br>vs control healthy teeth + MTA	**Histology**	**<u>Histological observation</u>**<br><u>Early time</u><br>1) Presence of regenerated tissues in the apical and middle thirds of the roots resembling a periodontal ligament tissue 2) inflammatory cell infiltration and dilated blood vessels decreasing<br><u>1 month</u><br>1) Mild to moderate periapical inflammation. omplete<br><u>3 months</u><br>1) Absence of inflammatory cell infiltration<br>2) Formation of mineralised hard and periodontal ligament-like tissues<br>3) Prominent hypercementoid proliferation with a fibrous scaffold in PDL<br>4) Maturation of the tissues in the periapical space which showed almost a complete maturation in PDL, alveolar bone with multiple accentuated reversal lines and osteons. 5) Osteoblastic rimming on the outer peripheries of bone

**Khazaei et al. 2020 (38)**	REP<br>Healthy immature premolar teeth	3 months	(Buccal fat) vs (BC + Buccal fat) + MTA	**Histology Immunohisto-chemistry and PCR**	**<u>Histological observation</u>**<br><u>Buccal fat</u><br>- 50% acute & chronic inflammation<br>- No complete calcified bridge<br>- No necrosis 75%<br>- DSPP, COL1A1, ALP are expressed<br><u>BC + Buccal Fat</u><br>- Chronic inflammation<br>- Complete calcified bridge: osteodentine<br>- No Necrosis<br>- DSPP, COL1A1, ALP, DMP1 are expressed
**Zaky et al. 2020 (39)**	REP<br>Infected immature premolar teeth	3 months	Collagen sponge vs PRF vs MTA	**Histology Immunohisto-chemistry 2D Radiographic**	**<u>Histological observation</u>**<br>1) Highly cellular through the length of the root canal with rich vascularity.<br>2) Inflammatory cells—manifested as an inflammatory plug: filled the apical foramen and bone and root resorption + no signs of regeneration or cellular<br>3) Non-inflamed roots also showed some intracanal mineralization with bone/cementum-like lamellar structures

**Mounir et al. 2019 (40)**	REP<br>Infected immature premolar teeth	1, 3, 6 months	Recombinant amelogenin protein RAP vs MTA	**Histology Immunohisto-chemistry**	**Histological observation**<br>1) Stem cells present: MSCs and SCAPs;<br>2) Regenerated tissues surrounding the root apices showed immune reactivity to Sox2, peripherin, CGRP and GFAP<br>3) Immunohistological evidence of the regeneration of peripherin neuronal intermediate filaments // GRP-reactive central nerves
**El-Tayeb et al. 2019 (41)**	REP<br>Infected immature premolar teeth	2 weeks; 1 & 2 months	Propolis vs MTA	**Radiographic Histopathology Histology**	**Radiographic observation**<br>1) Increase in root length and dentin thickness<br>2) Decrease in apical diameter<br>**Histologic observation**<br>Propolis<br>1) Formation of cementum like tissue on the inner aspect of the root dentin<br>2) Pulp like tissue with areas of osteoid<br>MTA<br>1) Formation of new layer of dentin like tissue<br>2) Fibrous connective tissue with islands of cementum like tissue inside the root canal space

**Huang et al. 2019 (42)**	REP<br>Infected immature incisor teeth	3 & 6 months	(Autologous pulp transplantation + BC) vs (BC) + MTA	**Radiographic**<br>**Histology**<br>**Immunohisto-chemistry**	**Radiographic analysis**<br>1) Decreased apical diameter<br>2) Increase in root wall thickness<br>3) Apical closure<br>4) Periapical healing<br>5) New hard tissue deposition<br>**Histological and immunohistochemical**<br>Autologous pulp transplantation<br>1) Canal walls had newly formed dentin-like tissue with dentinal tubules,<br>2) a lot of DSP+<br>3) Presence blood vessels<br>4) Presence fibrous tissue<br>Control group<br>1) Deposition of cementum-like tissue<br>2) Few DSP+<br>3) Presence blood vessels<br>4) Presence fibrous tissue

**El Halaby et al. 2020 (43)**	REP<br>Infected immature premolar teeth	1, 2, 3 months	(Blood clot) vs (17% EDTA + blood clot) vs (PRF) vs (17% EDTA + PRF) + MTA	**2D Radiography Histology**	**Histological & radiographic examination**<br>All groups<br>1) Increase in root length and thickness with signs of apical closure<br>BC<br>- Vital tissue infiltration along the apical two-thirds of the pulp<br>- Fibrous tissues and calcified structures inside the pulp space<br>- Partial hard tissue formation<br>- Signs of apical closure and mild inflammation<br>17% EDTA + blood clot<br>- Vital tissue infiltration along the pulp space,<br>- Fibrous tissue formation inside the pulp space<br>- Signs of complete hard tissue formation<br>- Apical closure and mild inflammatory cell infiltration<br>PRF<br>- Vital tissue infiltration along the pulp space<br>- Odontoblast-like cells with increased vascularity inside the pulp<br>- Complete hard tissue formation<br>- Signs of apical closure and no signs of inflammation<br>17% EDTA + PRF<br>- Vital tissue infiltration along the pulp space<br>- Odontoblast-like cells undergoing differentiation with increased vascularity inside the pulp space<br>- Complete hard tissue formation, signs of apical closure and no signs of inflammation

**El Kalla et al. 2019 (44)**	REP<br>Infected immature teeth + healthy immature premolar teeth	6 months	PRF +Portland cement vs Calcium Hydroxide	**Histology**	<u>**Histological observation**</u><br>Infected teeth<br>**PRF**<br>- Apical closure<br>- Hard tissue on internal walls<br>- Newly formed blood vessels<br>**Calcium hydroxide:** same but lower percentages.<br>Non-infected teeth<br>**PRF**<br>- Pulp closure<br>- Hard tissue on internal walls<br>- New pulp tissue formation<br>**Calcium hydroxide:** same but lower percentages.<br>root canal space
**Alqahtani et al. 2018 (45)**	REP<br>Infected immature molar + premolar teeth	2 months	Decellularized Swine Dental Pulp ECM vs BC	**Histology Immunohistochemistry Radiographic**	<u>**Histological & radiographic observation**</u><br><u>All groups</u><br>- Intracanal mineralization<br><u>DP-ECM</u><br>- DSP+ and CD31+<br>- pulp native microenvironment to recruit vascular cells

**Yoo et al. 2021 (46)**	REP<br>Infected immature premolar teeth	3 months	BC + ProRoot MTA	**Histology**<br>**Micro-CT**	**<u>Radiographic examination</u>**<br>- Radiographic repair of periapical radiolucency<br>- Continued root development<br>- Apical closure<br>**<u>Canal microenvironments histologically</u>**<br>- Devoid of bacteria contained<br>- New mineralized<br>- Pulp-like tissues
**Zarei et al. 2017 (47)**	REP<br>Infected immature premolar teeth closed and open apices	3 months	BC + Propolis vs BC + MTA vs BC + parafilm	**Immunohisto-chemistry**	**<u>Histological observation</u>**<br>**For MTA and Propolis**<br>- VEGF and factor VIII higher at 1 month than 3 months<br>**Expression of VEGF and factor VIII**<br>- Higher for Propolis than MTA group<br>- Higher in open apices in propolis group than closed<br>- Higher in closed apices in MTA group than open

**Londero et al. 2015 (48)**	REP<br>Infected immature premolar teeth	**7 months**	(BC) vs (BC + Gelfoam) + MTA	**Histology**	**Histological observation**<br>**BC**<br>- Mineralized tissue covering dentinal wall = cementum<br>- Increase in root length<br>- Ingrowth of the external cementum into the root canal space<br>**BC + Gelfoam**<br>- Mineralized tissue covering root canal space = bone like tissue<br>- Vascularized tissue<br>- Matrix with osteocytelike embedded cells<br>- Osteoblastlike cells on the surface<br>- Vital tissue formed in the root canal space = periodontal ligament<br>- Inflammatory cells: cervical third of the root canal next to MTA plug

**Stam-bolsky et al. 2016 (49)**	REP<br>Infected immature premolar teeth	**6 months**	Group A1: disinfection with sodium hypochlorite **(SH)** and **blood clot (BC) as scaffold;**<br>Group A2: disinfection with **SH** and **PRP as scaffold**;<br>Group B1: disinfection with **SH and (mTAP**) dressing for 15 days, and **BC blood clot as scaffold**;<br>Group B2: disinfection with **SH and mTAP** dressing, and **PRP as scaffold.**	**2D Radiography Histology**	**<u>Histological & radiographic examination</u>**<br><u>Experimental groups</u><br>- Different grades of arrested dentin development<br>- Gain of thickness of the apical third<br>- Bone-like or cementum-like tissues<br>**1) Vital tissue within canal spaces: in all group**<br>- More vital tissue in B2 group<br>2) **Hard-tissue deposition on radicular dentin walls: in all group**<br>- B2 group maximal percentage<br>**Apical closure**<br>- B2 group maximal percentage

**Wang et al. 2010 (50)**	REP<br>Infected immature premolar teeth	**3 months**	(BC) vs (BC + collagen type I) + MTA	**Histology**	**<u>Histological observation</u>**<br>- 43.9% of experimental teeth having hard tissue<br>- 29.3% having vital tissues in the canal spaces<br><u>BC</u><br>- Root thickness and length increased<br><u>BC + Collagen I</u><br>- Arrested dentin development<br>- Gain of thickness of the root = ingrowth of cementum-like<br>- No odontoblast layer<br>- Entire bridge = cementum -like<br>- Tissues resembling cementum, PDL and bone
**Zhang et al. 2014 (51)**	REP<br>Infected immature premolar teeth	**3 months**	PRP vs MTA	**2D radiography<br>Histology**	**<u>Histological & radiographic examination</u>**<br>- Apex closed PRP > BC<br>- Thickening of the canal wall with ingrowth of cellular cementum-like tissues PRP > BC<br>- Cementocyte-like cells were present in the newly formed tissues PRP > BC<br>- Pulp-like tissue formation in the canal space PRP > BC

**Palma et al. 2017 (52)**	REP<br>Infected immature premolar teeth	**3 months**	(BC) vs (HA:CS) vs (P:CS) + MTA	**Histologic Observation Histomorpho-metric analysis**	**Histological observation**<br>BC<br>- Resolution periapical lesion<br>- Root thickness and length<br>- Apical closure<br>- Bridge of cementum and dentin next to MTA<br>- Highly vascularized connective tissue<br>- Newly formed tissue intracanal = cementum<br>- Some specimen present odontoblastic layer<br>- Intracanal bone and alveolar bone in periapical area<br>HA:CS scaffold<br>- Resolution of periapical lesions (less than BC)<br>- Root thickness and length (same as BC)<br>- Apical closure (better than BC)<br>- Bridge of cellular cementum<br>- Highly vascularized connective tissue moderate inflammation<br>- Several areas of trabecular bone tissue<br>P:CS Scaffold<br>- Resolution of periapical lesions (less than BC and better HA:CS)<br>- Root thickness and length (better as BC and HA:CS)<br>- Apical closure (better than BC and HA:CS)<br>- Intracanal cementum next to dentin & apical third root<br>- Vascularized tissue without inflammation<br>- Bone tissues or osteoid like in small areas

**Saoud et al. 2015 (53)**	REP<br>Infected immature incisor teeth	**3 months**	BC + MTA	**Histology**	<u>**Histological observation**</u><br><u>Mineralized tissue</u><br>- Mineralized tissue next to MTA: Bone-like and cementum= repairing tissue.<br>-Hard tissue barrier was formed more apically<br>- Cells resembling elongated fibroblasts or mesenchymal cells with collagen fiber<br>- A concentration of inflammatory cells and scattered mineralized tissue nodules in the loose connective tissue<br><u>Soft tissue</u><br>- Loose connective tissue rich in blood vessels<br>- Without the presence of polarized columnar or flattened odontoblast-like cells<br>- No definite nerve fibers
**Zhu et al. 2014 (54)**	REP<br>Infected immature premolar teeth	**3 months**	(BC) vs (PRP) vs (Autologous DPSCs) vs (autologous DPSCs + PRP) + MTA	**Immunohistochemistry Histology**	<u>**Histological observation**</u><br>- Vital tissue BC > PRP, DPSCs + PRP > PRP<br>- Periostine + BC > DPSCs + PRP > PRP, DPSCs<br>2 types of mineralized tissue into canal space: cellular island and inner surface dentin walls.<br>- Nestin + None of them<br>- TRAP + BC > DPSCs + PRP, PRP, DPSCs<br>- DSP+ Into the tubules but no cells<br>- BSP and OCN Expression in Alveolar Bone and Root Canal Space

**Moradi et al. 2016 (55)**	REP<br>Infected immature premolar teeth	**3 months**	(BC + PRP) vs (BC) + MTA	**Immunohisto-chemistry Histology**	**<u>Histological observation</u>**<br><u>Vital tissue</u><br>- More without new vital tissue in both group than vital tissue<br>- More vital tissue MTA group than PRP group<br><u>Factor VIII expression</u><br>- More expression on both group after 1 month than 3 month<br>- 1month: MTA group > PRP group<br>- 3 months: MTA group < PRP group<br><u>VEGF expression</u><br>- More expression on both group after 1 month than 3 month<br>- 1month : MTA group < PRP group<br>- 3 months : MTA group < PRP group

**Fahmy et al. 2017 (56)**	REP Infected mature premolar teeth	**1 month**	(1) Double-antibiotic-paste/Blood clot, (2) Ciprofloxacin/collagen, (3) Double-antibiotic-paste/Collagen, (4) Modified Tri-antibiotic-paste /collagen, (5) Ciprofloxacin/Gelfoam, (6) Double-antibiotic-paste/Gelfoam, (7) Modified Triantibiotic- paste/Gelfoam.	**Histology Immunohistochemistry**	<u>**Histological observation**</u><br>- Different protocol are plausible for REP of mature canals with new intra-canal tissue<br>- Elarged apices to 0,6 mm for better REP procedure<br>- Neo-formed tissue better with collagen and Ciprofloxin or Diantibiotic<br>- Vascularity same as neo-formed tissue<br>- Hard tissue: Cementum like with collagen and Ciprofloxin or Di-antibiotic or no mineralized tissue<br>- Inflammation: every group has more mild inflammation except tri-antibiotic past / Gelfaom

**Khademi et al. 2014 (32)**	REP<br>Infected immature teeth vs Healthy immature premolar teeth	**3 and 6 months**	BC + MTA in inflamed vs vital pulp	**2D Radiography Histology**	**Radiographic observation**<br>- At 3 months : Better apical closure on vital pulp than inflamed pulp<br>- At 3 months : Better Thickness canal walls on vital pulp than inflamed pulp<br>- At 6 months : Better apical closure for inflamed pulp<br>- At 6 month : Same results on Thickness canal walls for inflamed pulp<br>- At 6 months : Better healing periapical lesion for inflamed pulp<br>**Histologic observation**<br>- Inflammation in periapical tissue : mild for all groups<br>- Nature of coronally proliferated tissue : Granulation for all groups<br>- New hard tissue is distinct for most of the specimen of each group or Limited on dentinal walls.

**Thibodeau et al. 2007 (57)**	REP<br>Infected immature premolar teeth	**3 months**	(BC) vs (collagen type I) vs<br>(BC + Collagen type I) + MTA	**2D Radiography Histology**	**<u>Radiographic evaluation</u>**<br>Thickening of Root Canal Walls<br>- Collagen + BC > BC > Collagen<br>Apical Closure<br>- BC > Collagen > Collagen + BC<br>Healing of Periapical Radiolucency's<br>- BC > Collagen + BC > Collagen<br>**<u>Histological observation</u>**<br>Hard-Tissue Deposition on Radicular Dentin Walls<br>- Collagen + BC > BC > collagen<br>Histological apical closure<br>- Collagen + BC = BC > Collagen<br>Histological Vital Tissue Within Canal Spaces<br>- Collagen + BC = BC > Collagen

**Rodríguez-Benítez et al. 2015 (58)**	REP<br>Infected immature premolar teeth	**6 months**	Gp 1: NaOCl/Blood Clot<br>Gp 2: NaOCl/PRP<br>Gp 3: NaOCl/TAP/Blood Clot<br>Gp 4: NaOCl/TAP/PRP	**2D Radiography**	**Radiographic examination**<br>**Radiographic Healing of periapical radiolucency**<br>Gp 4 > Gp 3> Gp2 > Gp1<br>**Increase in Root Thickness**<br>Gp 4 > Gp 3> Gp2 = Gp1<br>**Radiographic Apical Closure**<br>Gp 4 > Gp 3> Gp2 = Gp1
**Zhou et al. 2017 (59)**	REP<br>Infected immature premolar teeth	**3 months**	(BC) vs (BC + PRF) + MTA	**2D Radiography Histology**	**Radiographic observation**<br>- Periapical healing for all groups<br>- Increasing root length and thickness are almost the same for both groups<br>- Better resistance fracture for BC + PRF > BC alone.<br>**Histologic observation**<br>- Newly formed mineralized tissue with Cementocyte-like cells at the apex and along the internal canal wall for both groups<br>- Bone like structure in canal space almost for all specimen for both groups

**El Ashry et al. 2016 (66)**	REP<br>Infected immature premolar teeth	**1,5 to 3 months**	(BC) vs<br>(BC + collagen) vs<br>(BC + EDTA) vs<br>(BC + EDTA + collagen)<br>vs<br>(BC + MTAD) vs<br>(BC + MTAD + colla-gen)<br><br>+ MTA	**2D Radiography Histology**	**<u>Radiographic examination</u>**<br>- All experimental groups increase in root length and thickness<br>- All experimental groups decrease in apical diameter among<br>**<u>Histological observation</u>**<br>- More bone resorption for blood clot + collagen, blood clot + EDTA + collagen, blood clot + MTAD + collagen<br>- New pulp space tissue : resembled the periodontal connective tissue with various inflammatory cells and angiogenesis<br>- New hard tissue in the canal space : cemotoide like tissue with different shape and Thickness

**Da Silva et al. 2010 (61)**	REP<br>Infected immature premolar teeth	**4 weeks**	Group 1: apical negative pressure irrigation<br>vs<br>Group 2: conventional irrigation plus Tri-antibiotic intracanal dressing + BC + MTA	**Histology**	**<u>Histological observation</u>**<br><u>Group 1</u><br>- Dense, well-structured ingrowth connective tissue<br>- Young fibroblasts<br>- Rich blood vessels<br>- Mineralized tissue along canal walls<br>*Periapical region :*<br>- intense revascularization<br>- advanced repair process<br>- absence of inflammatory cells<br><u>Group 2</u><br>- Connective tissue ingrowth into the root canal<br>- Less exuberant vessels and nerve bundles than group 1<br>- connective tissue ingrowth reached the middle and apical canal third<br>- Mild inflammation<br>- Beginning of the formation of mineralized tissue along the root canal walls<br>*Periapical region :*<br>- intense revascularization<br>- advanced repair process<br>- few of inflammatory cells (macrophages, plasmocytes)

**Pagliarin et al. 2016 (62)**	REP<br>Infected immature premolar teeth	**7 months**	(Triple antibiotics) vs (propolis) + MTA	**Histology**	**<u>Histological observation</u>**<br>New mineralized tissue formation on dentinal walls and in root canal space<br>- Cementum-like mineralization for both groups<br>Presence of vital tissue in root canal space<br>- Periodontal-like for both groups<br>Apical extension of root<br>- Present for both groups<br>Inflammatory reaction<br>- Moderate > severe > mild = absent for propolis<br>- Severe > Mild > absent
**Zhu et al. 2012 (63)**	REP<br>Infected immature premolar teeth	**3 months**	1) Blood clot vs<br>2) autologous DPSC vs<br>3) PRP vs<br>4) BC +DPSCs +PRP<br>+ MTA	**Histology**	**<u>Histological observation</u>**<br>- Vital Tissues in the Canal Space<br>- Vital full-length BC > PRP > Mixture > DPSCs<br>- Vital half-length DPSCs = PRP = mixture<br>- Necrotic DPSCs > PRP > Mixture<br>- Close to MTA = Osteoid hard tissue<br>- Cementum-like tissue and periodontal ligament–like tissue along the dentinal wall<br>- Rare specimen with bone-like tissue

**Sheep model**					
**Altaii et al. 2017 (64)**	REP<br>Infected immature incisor teeth	6 months	BC + MTA	**Micro-CT**<br>**Histology**	**<u>Radiographic examination</u>**<br>- Increasing in root length<br>- Increasing root wall thickness<br>- Decreasing root diameters<br>**<u>Histological observation</u>**<br>Root maturation and thickening of the walls<br>- Hard tissue deposition in all experimental teeth.<br>- Hard tissues in the apical portion of the root were more developed than in the coronal portion
**Ferret model**					

**Alexander et al. 2020 (22)**	REP<br>Healthy immature canine teeth	3 months	(SynOss Putty) vs (BC)+ MTA	**3D Radiography Histology**	**<u>Histologic observation</u>**<br><u>BC</u><br>- Root canal thin<br>- Apices were closed<br>- No odontoblast layer<br>root canal space filled bone-like tissue<br><u>Synoss Putty</u><br>- Root canal thin<br>- Apices were closed<br>- No odontoblast layer<br>- No signs of new tissue formation<br>- Intracanal and periapical inflammation<br>**<u>Radiologic observation</u>**<br>Apical Diameter: No significant difference between groups

**Tora-binejad et al. 2018 (23)**	REP with residual incisors dental pulp tissue	3 months	- complete pulp extirpation<br>- pulp amputation to 1–2 mm short of the radiographic apex<br>- pulp amputation 2–4 mm from the radiographic apex<br>BC + MTA	**3D Radiography Histology**	**<u>Histological & radiographic examination</u>**<br><u>Complete pulp extirpation teeth</u><br>- Presence of bone inside the root canal<br><u>Pulp amputation 1–4 mm</u><br>- Presence hard tissue bridges<br><u>All groups present</u><br>- Apical closure<br>- Normal tissue<br>- Absence of intracanal/ periapical inflammation<br>- Wall thickening increased

**Verma et al. 2017 (26)**	REP<br>Infected immature canine teeth	3 months	(BC) vs (DPSCs + hydrogel scaffold) + MTA	**2D Radiography**<br>**Histology**<br>**Bacteriology**	**<u>Radiographic Examination</u>**<br>- Increase in root wall thickness<br>- Increase canal width<br>- Decrease in canal diameter<br>- Increase in root length<br>- Presence/absence of radiographic lesion<br>--> No significant difference between two groups<br><br>**<u>Histologic observation</u>**<br>BC group : 2 distinct histologic patterns of tissue organization<br>- Loosely organized connective tissue<br>- Newly deposited mineralized tissue<br>- Some specimen osteodentin mixed with loose connective tissue (both groups)<br>- Specimen without radiographic growth had inside de canal space bony island<br>No radiographic growth = residual bacteria : significant

**Tora-binejad et al. 2015(24)**	REP<br>Infected immature canine teeth	3 months	(BC + Gelfoam) vs (PRP) + MTA	**Histology**	**<u>Histological observation</u>**<br>- Bone or cementum mixed with connective tissues on apical third for some PRP & BS + Gelfoam<br>- Resolution of a periapical lesion + hard tissue ingrowth for all groups<br>- Connective tissue contained vessels and fibroblasts.<br>- Coronal to the ingrowth of hard tissue and connective tissue, granulation tissue and inflammatory cells<br>- Thickening of the root walls: bone-like and cementum-like tissue<br>- PRP group showed a more prominent ingrowth of hard tissue in the apex than BC/Gelfoam group.
**Tora-binejad et al. 2014 (25)**	REP<br>Infected immature canine teeth	3 months	(BC) vs (BC + PRP) + MTA	**Histology**	**<u>Histologic observation</u>**<br>- Ingrowth of bonelike tissue from the apical region extending to the coronal third of the root for almost all specimen of both group<br>- Bonelike tissue mixed with connective tissue + blood vessels and fibroblasts<br>- Most of the specimen of both group showed inflammatory cells: macrophages and lymphocytes<br>- No significant difference between groups.

**Mice Model**					
**Yadlapati et al. 2017 (21)**	Ectopic	1,5 months	VEGF-loaded fiber + root fragment + MTA	**Histology**	**Histologic evaluation**<br>Implanted VEGF<br>- New vessel formation<br>- Mild inflammation: macrophages presence around the implanted fibers<br>- Decrease of inflammation<br>- Increase of new blood vessels
**Li et al. 2016 (20)**	Ectopic	2 months	Root tooth + (DPSC)<br>vs<br>(VEGF-loaded HG-MS)<br>vs<br>(VEGF-loaded HG-MS + DPSC)<br>+ MTA	**Histology Immunohisto-chemistry**	**Histologic observation**<br>- Soft tissue formation in the roots: VEGF-loaded HG-MS<br>- Pulp-like tissue: DPSCs group<br>DPSCs differentiated into odontoblast-like cells and aligned with the existing tubular root dentin.<br>- regenerate tissue fulfilled whole root and reach coronal third: VEGF-loaded HG-MS/DPSC group<br>vWF and CD31<br>VEGF-loaded HG-MS/DPSC group > VEGF-loaded HG-MS<br>Nestin and DSP<br>DPSC and VEGF-loaded HG-MS/DPSC groups > VEGF-loaded HG-MS<br>Human mitochondrial<br>- Pulp-like and odontoblast-like cells were marked strongly
**Rat Model**					

**Edanami et al. 2020 (31)**	REP<br>Immature molar teeth	1 month	BC + MTA on residual healthy pulp	**Histology<br>Immunohisto-chemistry**	**<u>Histologic observation</u>**<br>Teeth with 0.81–0.91 mm of remnant pulp healed<br>- Pulp like tissue : dentin and osteodentin<br>Teeth with 0.60–0.63 mm of remnant pulp healed<br>- Pulp like tissue : dentin and osteodentin<br>Teeth with 0.13–0.43 mm of remnant pulp healed<br>- (PDL)-like tissue, dentin and osteodentin, and cementum-like<br>Teeth with disorganization of pulp and apical tissues at 0.150.38 mm beyond the root apex healed<br>- PDL-like, cementum-like, dentin associated mineralized tissue and intra-canal bone.
**Wen et al. 2020 (16)**	Ectopic	3 months	Tooth slice + rBMSC (collagen scoffold) + iRoot BP<br>rBMSC (collagen scoffold)<br>The collagen	**Histology<br>Immunohisto-chemistry**	**<u>Histological observation</u>**<br>- Analysis on neovascularization : new blood vessels were greater in BP1/3 than in M1/3 and D1/3<br>- DSP : immunoreactivity in all pulp-like tissues (rBMSC gp)<br>- PGP9,5 : labeling in soft tissue intense in the nerve-like cells<br>- CD31 suggested angiogenesis in the new pulp tissues (rBMSC+ iRoot BP significant)<br>- rBMSC : pulp like tissue filled the tooth slice + regeneration of blood vessels<br>- Collagen : fibrous tissue inside and outside the tooth

**Chen et al. 2021 (30)**	REP<br>Mature Healthy<br>Molar teeth	1 month	L-sEVs<br>N-sEVs<br>BMSCs<br>BMSCs + L-sEVs<br>BMSCs + N-sEVs	**Histology**<br>**Immunohisto-chemistry**	**<u>Histologic observation</u>**<br>- N-sEVs and L-sEVs modulate BMSC proliferation, migration, angiogenesis, and differentiation.<br>- Both enhanced the structure of the regenerated tissue closer to that of a normal dental pulp in vivo.<br>- L-sEVs had a more significant effect than N-sEVs.<br>- L-sEVs more blood vessels, loose connectives tissue than N-sEVs<br>- Collagen fibers arranged along the dentin wall<br>- More TGF-β expressed L-sEVs than N-sEVs
**Na-beshima et al. 2018 (29)**	REP<br>Mature Healthy<br>Molar teeth	2 months	**Group I** : BC vs **Group II** : BC + 5 μL of VEGF + light-curing glass-ionomer cement	**Histology**<br>**Immunohisto-chemistry**	**<u>Histologic observation</u>**<br>- Formation of loosen connective tissue without presence of odontoblasts in all specimens<br>- Necrotic tissue surrounded by collagen fibers in all, although necrotic spots were more evident in group 1 than in group 2<br>- Formation of highly cellular and well-developed tissue with presence of fibroblasts and functional vessels filled with blood cells<br>- Newly formed tissue in most of specimens from both groups and thirds

**Gomes-Filho et al. 2012 (19)**	Ectopic	7, 15 days and 1, 2, 3 months after implantation	In polyethylene tubes: TAP (antibiotic mixture) vs CHP calcium hydroxide powder with saline	**Histology**	**Histologic observation**<br>- Day 7 and 15, moderate chronic inflammatory cell infiltration by lymphocytes and macrophages was found in a fibrous capsule for TAP ,CHP, control<br>- Intensity of inflammation reduced on days 30, 60, and 90<br>- Thin fibrous capsule developed near the tubes for TAP, CHP & control<br>- Dystrophic mineralization was only observed with calcium hydroxide.<br>- Day 7 and 15, thin fibrous capsule formation and moderate cell inflammatory infiltration by lymphocytes and macrophages was observed with macrogol/propylene glycol<br>- Mild inflammatory cell infiltration and a reduction in the thickness of the fibrous capsule was evident from day 30

**Moreira et al. 2017 (28)**	REP<br>Mature Healthy Molar teeth	1 month	Blood clot + PBM (photobiomodulation)	**Histology Immunohisto-chemistry**	**Histologic observation**<br>Group control:<br>No tissue formation<br>Group PBM:<br>- Root canal filled with immature connective tissue fewer and thinner collagen fibers than the originally preserved dental pulp<br>- Blood vessels exhibiting SMA-positive cells in their walls<br>- S-100-positive cells underneath the odontoblast-like cell layer<br>- STRO-1-positive cells in perivascular niches
**Ruang-sawasdi et al. 2014 (18)**	Ectopic	3 months	human teeth roots + fibrin gel	**Histology Immunohisto-chemistry**	**Histologic observation**<br>Fibrin gel :<br>- Improve tissue formation<br>- 43% of total root filled by new tissue<br>- Development small blood vessels<br>- New tissue looks like normal pulp with cell-rich zone, cell-free zone, and odontoblast layer<br>- Odontoblast-like cells found close to the dentinal wall<br>- BSP+ areas were in the cells integrated to the dentinal wall<br>- DSP+ areas in predentin in roots filled with fibrin gel, while they were absent in the formerly empty root canals.

**Scarparo et al. 2011 (27)**	REP<br>Infected immature teeth vs vital pulp	3,6,9 weeks	triple antibiotic paste+ silver amalgam	**Radiographic**<br>**Histology**	**<u>Radiographic examination</u>**<br>- Better increasing of root length with polyATB after 3- and 6-weeks post-treatment and significantly different from control.<br>- Better decreasing root canal width with polyATB after 3- and 6-weeks post-treatment and significantly different from control.<br>- Better decreasing Periapical lesion with polyATB after 3- and 6-weeks post-treatment than control.<br>**<u>Histological observation</u>**<br>- Induction of necrosis arrested root formation<br>- Variable inflammatory response to treatment<br>- Half of the roots showed formation of a cementum-like tissue on its apical portion<br>- External new formed cementum

**Rabbit model**					
**El-Backly et al. 2008 (17)**	Ectopic	12 days	rDPSCs+spoly(lactic-co-glycolic acid) polymers scaffolds	**Histology**	**Histological observation**<br>Group I:<br>- No sign of a fibrous tissue capsule<br>- Lot of newly forming blood vessels and budding capillaries<br>- Increased cellularity in the form of numerous stellate-shaped or fibroblast-like cells.<br>Group II:<br>- Capsule seems to contain fibrous tissue<br>- Columnar cells arranged in tubular-like structures<br>parallel tubules like a dentine-like appearance

Table S2: Animal models for Ectopic and Rep techniques.

Author/ Years	Clinical context	Follow-up	Materials or REP procedure	Assessment	Results
**Human model**					
**Jha et al. 2019 (134)**	REP<br>Periapical infection with completely formed roots	6,12,18 months	SealBio vs Obturation	**clinical signs PAI**	**<u>Clinical tests</u>**<br>- PAI score diminution<br>- Presence or absence of clinical signs and symptoms<br>- Healed—combined radiographic and clinical normalcy<br>Healing—reducing radiolucency with clinical normalcy<br>Diseased—if the radiolucency persisted without change with or without clinical normalcy.<br>Both groups showed equally favorable outcomes at the end of 18 months without any statistically significant differences.<br>**<u>Success</u>**<br>TAP + irrigation = sterile roots<br>- Maintenance of a sterilized canal system with the help of a coronal seal<br>- Use of negative pressure irrigation system.<br>- Placement of triple antibiotic paste in the canals which reduces the bacterial load tremendously by penetrating the dentinal tubules

**Aly et al. 2019 (85)**	REP<br>Necrotic immature permanent anterior incisor teeth	3, 6, 9, and 12 months.	BC + MTA vs BC + Biodentine	**Clinical tests**<br>**2D Radiography**	**Clinical tests**<br>- Complete regression of signs and symptoms: pain, swelling, and sinus or fistula 100% with Biodentine and 95% MTA.<br>- Discoloration: Biodentine 8% / MTA 59%<br>**Radiological analysis**<br>- Root length increasing but no significant Biodentine 5,64% and MTA 5,02%.<br>**Fail:**<br>- Presence of pain on biting and pain on percussion at 12-month follow-up<br>- Might be due to the increased bacterial growth in the dentinal tubules
**Brizuela et al. 2020 (115)**	REP<br>Periapical lesion<br>Mature permanent incisor, premolar and canine teeth	6-12 months	UC-MSCs + Collagen membrane + Biodentine vs gutta percha (ENDO)	**Clinical tests**<br>**3D Radiography**	**Clinical tests**<br>- Thermal and Electrical Tests REP > ENDO<br>**Radiographic analysis**<br>Dimensions of the Apical Lesions: diminution in both group but better with ENDO technique

**Arslan et al. 2019 (86)**	REP<br>Necrotic Mature Teeth incisors with Periapical lesion	12 months	BC + MTA vs endodontic treatment	**Clinical tests Radiographic analysis 2D with image J with scoring (measure the change of lesion size between pre- and postoperative radiographs)**	**Clinical tests**<br>- Absence of clinical signs and symptoms<br>**Success treatment**: **92, 3%** favorable, which 15% respond positively to electrical vitality, test.<br>**Radiographic examination**<br>80% radiographic outcomes favorable.<br>- No statistical significant difference between groups terms of the pre-operative radiographic lesion score<br>- Absence and reduction of the radiolucency were observed in 8**5% of CRCT-treated** teeth compared with **92.4% of REP-treated teeth**<br>- Reduction of the periapical lesion **25% CRCT-treated / 46.2% REP**<br>- The number of discolored teeth **REP group 38.5**<br>**Fail**<br>- remnants of microbial irritants and necrotic pulp tissues. None of the teeth with failure had pain, percussion, or palpation pain: 6/46
**Ragab et al. 2019 (87)**	REP<br>Necrotic Immature Permanent incisors	6 months to 12 months follow up	With PRF vs PRF + MTA	**2D Radiography**	Radiographic examination<br>- Root length increase through the follow up period **with PRF 8,8% to 12,3%** or **without 7,7% to 14,8%.-** Apical barriers were observed in 5 cases in-group (A) and 7 cases in-group **(**B). Observation calcific barrier: No PRF > PRF<br>- Decrease in the perimeter of the periapical radiolucency. Both groups decrease perimeter<br>- No statistically significant differences between the 2 groups.

**El-Kateb et al. 2020 (114)**	REP<br>Mature necrotic incisors teeth with periapical lesions	1, 3, 6, 9, and 12 months	Protaper Next files X3 test vs X5 control<br><br>BC + Biodentine	**MRI: regenerate pulp-like tissue Radiographic evaluation: assess periapical healing. Clinical tests**	**Clinical tests**<br>- No pain, no sensitivity to percussion, or swelling 100%<br>- Cold test response positive for 77,8% Biodentine group and 88,9% for control.<br>- EPT: 66,7% Biodentine group and 88,9% control group.<br>- 83% of all the cases treated responded positively to cold after 12 months, whereas 77% of all cases responded positively to the EPT.<br>**MRI**<br>- The significant decrease in the SI to reach the normal contralateral teeth<br>- More organized tissue in the canal, dentin deposition and mineralization<br>**2D radiographic examination**<br>- Complete healing of the periapical lesion on both groups
**Sallam et al. 2020 (6)**	REP<br>Necrotic immature permanent premolar, molar, and incisors teeth	3,6,9 months	Contamined teeth: PRF vs Calcium Hydroxide<br>Non contamined PRF vs Calcium Hydroxide	**Radiographic examination and measurement**	**Clinical Success** 100%<br>- No pain either spontaneous or on biting recorded as present or absent by asking the patient.<br>- No tenderness to pressure or percussion<br>- No tenderness to palpation of adjacent soft tissues.<br>- No sinus tract or swelling in adjacent soft tissues.<br>**Radiographic examination**<br>- Significant difference in all intervals regarding root length in PRF subgroups and calcium hydroxide.<br>- Increase in root length and thickness,<br>- Decreased diameter of apical foramen

**Rizk et al. 2020 (126)**	REP<br>- permanent necrotic maxillary central incisors with incomplete root<br>- with or without periapical lesions<br>- pulp involvement due to caries or trauma	3, 6, 9 and 12 months	PRF + MTA vs PRP + MTA	**Clinical tests Radiographic analysis and measurement**	**<u>Clinical tests</u>**<br>- resolution of pain, swelling, mobility and sinus/fistula = **100% success**<br>- Discoloration: PRF > PRP<br>- All REP: **0%** no respond to pulp sensibility tests (thermal (cold/heat), and electric pulp tester)<br>**<u>Radiographic analysis</u>**<br>- Increase root length PRP > PRF<br>- Increase root width PRP < PRF<br>- Increase in bone density PRP > PRF<br>- Decrease in apical diameter PRP > PRF<br>But no statistical significant differences<br>No sign of calcification/obliteration.
**Jiang et al. 2017 (88)**	REP<br>Non-vital<br>Immature premolar teeth	3, 6, 9 and 12 months	Bio-gide collagen on blood clot + MTA	**Clinical tests Radiographic evaluation by measurement**	**<u>Clinical tests</u>**<br>- Complete resolution of signs and symptoms.<br>- EPT, n (%) Control 18%/ test 33%<br>**<u>Radiographic analysis</u>**<br>- Increase in root length, n (%) Control 100% / Test 100%<br>- Increase in dentin wall thickness in the apical third of the root, n (%) Control 91% / test 86%<br>- Increase in dentin wall thickness in the middle third of the root, n (%) Control 55% / 100% test<br>- Narrowing of apical foramen width, n (%) 91% control/ 100% test<br>- Discoloration, n (%) 64% control / 71% test Calcification, n (%) 55% control /48% test

**ElSh-eshtawy et al. 2020 (7)**	REP Immature Permanent anterior incisors teeth with necrotic pulps	3, 6, 9 and 12 months	(BC+ PRP) vs (BC) + MTA	**Clinical tests Radiographic evaluation by measurement**	**<u>Clinical tests</u>**<br>- thermal and electric pulp testing: No response<br>**<u>Radiographic examination</u>**<br>- Root length: BC > PRP<br>- Dentine wall thickness BC < PRP<br>- Apical foramen width BC > PRP<br>- Radiographic root area BC > PRP<br>3/31 fail cases because of reinfection
**Rizk et al. 2019 (89)**	REP Necrotic Immature Permanent incisors teeth	3, 6, 9, and 12 months	(PRP) vs (BC) + MTA	**Clinical tests Radiographic evaluation by measurement with image J**	**<u>Clinical tests</u>**<br>- resolution of pain, swelling, mobility, and sinus/fistula = 100% success<br>- 100% no response to pulp test<br>**<u>Radiographic outcome</u>**<br>- Root length PRP > BC<br>- Root thickness PRP > BC<br>- Increase in bone density PRP > BC<br>- Decrease in apical diameter PRP > BC<br>No difference significant

**de-Jesus-Soares et al. 2020 (128)**	REP<br>Necrotic immature incisor teeth	21 days	Different treatments interappointment medication<br>Root canal samples were taken before treatment (S1), after 6% NaOCl irrigation (S2), and after interappointment medication (S3).	**Radiographic evaluation Bacteria Quantification by qPCR**	**Clinical tests**<br>- Spontaneous pain 0%<br>- Pulp sensibility (cold test) 0%<br>- Pulp sensibility (electric test) 27% (2 cases TAP group/ 1 case CHP)<br>- Pain on percussion 0%<br>- Pain on palpation 0%<br>- Sinus tract 0%<br>- Abscess 0%<br>**Radiographic examination**<br>- Periapical lesion 0%<br>- Root canal thickness 73% (TAP 60%/ CHP 83%)<br>- Root length increase 82% (TAP 60%/ CHP 100%)<br>better CHP > TAP<br>- Root resorption 0%<br>- Apical closure (TAP 80%/ CHP 100%)<br>3 cases failed before the 1st review appointment and 1 recurrent trauma.
**Ramachandran et al. 2020 (65)**	REP<br>Immature permanent incisors teeth with necrotic pulps and apical periodontitis	6 & 12 months	(BC) vs (BC + PRP) + MTA	**2D Radiography**	**Clinical tests**<br>- None of them responded to vitality testing on the follow-up visits.<br>- None of the cases had increased periapical radiolucency<br>**Radiographic examination**<br>- BC group: RRA increased 3% to 9,8% at 12 months<br>- PRP group: RRA increased 4,5% to 9,6% at 12 months<br>4 years after 2 patients creation calcification barrier

**Botero et al. 2017 (90)**	REP<br>Pulp necrosis on an immature permanent premolar and molar tooth or patients with at least 1 open apex of 1 mm or more in diameter	3,12,24 months	(BC + Collacote piece + MTA) vs Calcium Hydroxide	**Clinical tests**<br>**2D Radiographic**	**No results on clinical or radiographic success.**<br>Collacote piece + MTA group: success rate of 71% 100% success for delayed group / CaOH2
**Shivashankar et al. 2017 (91)**	REP<br>Necrotic immature permanent teeth & trauma and/ or caries	3rd, 6th, 9th and 12th months	(PRF) vs (PRP) vs (BC) + MTA	**Clinical examination**<br>**Radiographic evaluation by measurement with image J**	**Clinical tests**<br>- Absence of pain, inflammation or swelling<br>- 2 failed with PAI= 4 even asymptomatic<br>- PAI score 1 or 2<br>**Radiographic examination**<br>- Complete healing majority of patient<br>- Root Lengthening and Lateral Wall Thickness better PRF > PRP > BC<br>- Presence of a normal periodontal ligament space<br>Vitality responses 15% PRF, 13,3% BC, 15,8%.<br>PAI score 3,4,5 = failure: 10/118

**Bezgin et al. 2015 (69)**	REP Necrotic single-rooted immature incisor and premolar teeth	3 months and 18 months	(BC) vs (PRP + BC) + MTA	**Clinical examination Radiographic evaluation by measurement with image J**	**Clinical tests**<br>- Asymptomatic<br>- Positive response to vitality testing (50% PRP / 20% BC)<br>**Radiographic examination**<br>- Complete apical closure<br>- Increase in RRA<br>- BC group exhibited a mean increase of 12.6%<br>- 9.86% in the PRP group<br>Difference between groups was statistically insignificant<br>- No discoloration in relation to the triple antibiotic paste/<br>- Crown discoloration caused by MTA (60%)<br>Successful outcomes PRP: 50% /// 20% BC
**Nagy et al. 2014 (70)**	REP Immature permanent incisor teeth with necrotic pulps	3, 6, 12, 24, months	MTA (apexification) vs (BC + MTA) vs (BC + hydrogel with FGF+ MTA)	**Clinical examination Radiographic evaluation by measurement with image J**	**Clinical & radiographic success**<br>MTA, REG, and FGF groups were 100%, 90%, and 80%<br>**Radiographic examination**<br>1) Increase in Root Length: YES for all groups<br>- No significant difference between the REG and FGF<br>- FGF > REG.<br>2) Increase in Root Thickness: YES<br>- No significant difference between the REG and FGF<br>- FGF < REG.<br>3) Decrease in Apical Diameter. YES<br>- MTA group was significantly different from the REG and FGF 4) Periapical Bone Density<br>- Significant improvement in bone density all groups.<br>- No significant difference was found between all groups through the whole follow-up period<br>3/29 fail because symptomatic

**Alagl et al. 2017 (71)**	REP<br>Non-vital immature permanent premolar and incisor teeth	5-12 months	(BC) vs (PRP +BC)+ MTA	**Clinical test**<br>**2D radiography**<br>**3D radiography**	**<u>Clinical tests</u>**<br>- Resolution of signs and symptoms all patients - Sensitivity tests: 80% PRP / 40% BC<br>**<u>Radiographic examination</u>**<br>- Decreased lesion size both groups<br>- Increased bone density both groups<br>- Increased Root length both groups PRP > BC<br>- Complete apical closure: PRP > BCMean difference in the root length was found to be statistically significant
**Nagata et al. 2014 (129)**	REP<br>Immature incisor teeth diagnosed with pulp necrosis caused by hard tissue dental trauma and/or some severe luxation	9-19 months	Intracanal medicament: TAP vs calcium hydroxide and 2% CHX gel	**Microbiological and PCR**<br>**Clinical tests**<br>**Radiographic Examination**	**<u>Microbiological analysis</u>**<br>- Progressive disinfection help to destroy every bacteria<br>- Bacterial reduction: irrigation solutions.<br>**<u>Clinical tests</u>**<br>- Complete reduction of spontaneous pain, pain on percussion, pain on palpation, fistula, and abscess<br>- No pulp response<br>**<u>Radiographic examination</u>**<br>- No info
**Narang et al. 2015 (122)**	REP<br>Necrotic immature permanent teeth with or without associated apical periodontitis	6-18 months	MTA apexification vs (BC) vs (PRF clot) vs (PRP + collagen) + resin-modified glass ionomer cement	**Clinical tests**<br>**2D Radiography**	**<u>Clinical tests</u>**<br>- Asymptomatic with no tenderness on either percussion or palpation.<br>- Swelling and sinus had resolved completely.<br>**<u>Radiographic analysis</u>**<br>- Periapical healing PRF 98% > PRP 80% > BC 60% > MTA 58%<br>- Apical closure: 66.67% BC, 40% PRF, and 60% PRP showed good apical closure.<br>- Root lengthening 99% PRF > 60% PRP & BC<br>- Dentinal wall thickening: 60% of the cases in Group PRF; good

					results were obtained in 50% of cases in Group BC and 20% of cases in Group PRP
**Nakashima et al. 2017 (123)**	REP<br>Mature incisor/molar permanent teeth	1, 2, 4, 12, 24,28,32 weeks	MDPSCs were transplanted with granulocyte colony-stimulating factor (G-CSF) in atelocollagen into pulpectomized teeth.	**Clinical Tests Radiographic Examination MRI radiography**	**Clinical tests**<br>Negative on<br>- Cold/hot pain<br>- Percussion pain<br>- Tenderness<br>Positive<br>- Electric pulp test<br>**Radiographic examination**<br>- 24/28 weeks : obliteration of the enlarged apical portion following pulpectomy in three cases (patients 1, 3, and 5)
**Ibrahim et al. 2021 (116)**	REP<br>necrotic upper central incisor mature, pulp necrosis associated with apical periodontitis.	3, 6, 9 and 12 months	iPRF + Biodentine	**Clinical tests 3D radiography**	**Clinical tests**<br>- no pain, swelling or fistula formation<br>**Radiographic examination**<br>- Periapical lesion decrease<br>iPRF > control

**Cerqueira-Neto et al.2021 (132)**	REP Traumatized Immature necrotic maxillary incisor Permanent Teeth with pulp necrosis and periapical lesion	24 months	Single visit: BC + scaffold 2 visits : BC + scaffold + coltosol	**Clinical tests**<br>**2D Radiography**	**<u>Clinical tests</u>**<br>- No failure clinical<br>- Pulp sensibility: 9% vs 33,3%<br>**<u>Radiographic examination</u>**<br>- Interappointment 82% vs Single visit 100%<br>- Root development evaluation: 45,5% vs 66,7%<br>- Decrease of apical diameter ID < SV<br>- Increase of root width ID < SV<br>- Increase in root length ID < SV
**El Ashiry et al. 2016 (60)**	REP immature permanent teeth with necrotic dental pulps.	2 weeks, 6, 12, and 24 months	BC + MTA	**Clinical tests**<br>**2D Radiography**	**<u>Clinical criteria</u>**<br>- Patient were asymptomatic<br>- No pulp sensibility thermal and percussion<br>- No swelling<br>**<u>Radiographic analysis</u>**<br>- Increased root length increased<br>- Root canal wall thickness.<br>- Resolution of periapical lesion

**Nosrat et al. 2019 (121)**	REP<br>Immature healthy premolar maxillary and mandibular	2.5–7.5 months	Blood clot + SynOss Putty or SynOss only	**Teeth extraction**<br>**2D Radiography**<br>**Histology**	**Clinical tests**<br>- Normal mobility, probing depths<br>- No signs or symptoms of endodontic disease<br>- Negatively to sensibility tests except for 1 patient.(Synoss putty + BC)<br>**Radiographic analysis**<br>- All extracted teeth had immature apices<br>- Comparison of the MTA level between these teeth showed well-controlled placement in all SynOss Putty cases versus poor control<br>**Histological observation**<br>- New tissue formation<br>- No resorption, inflammation in the newly formed intracanal tissues<br>- Normal connective tissue with fibroblasts, and blood vessels.<br>- Newly formed mineralized tissue<br>2 types of newly formed mineralized tissue<br>1) Cementum-like tissue near the scaffold and dentinal wall<br>2) Bonelike islands in the root canal space and deposition of a mineralized cementum-like tissue on the dentinal walls.
**Lovelace et al. 2011 (136)**	REP<br>immature permanent incisors & premolar maxillary or mandibular single-rooted immature tooth with open apices	1 month	Collect an intracanal blood sample after BC.	**Biochemical tests**<br>**PCR to identify stem cells in the canal**	**PCR finding**<br>- Indicated the significant accumulation of transcripts for the stem cell markers CD73 and CD105 compared to systemic blood.<br>- No change in expression of the differentiation markers ALK-P, DSPP, ZBTB16, and CD14

**Meschi et al. 2021 (124)**	REP Immature Permanent incisors and premolar teeth with necrotic pulp	3, 6, 12, 24, and 36 months	BC + Collaplug + Portland cement vs BC + LPRF + Portland cement	**2D/ 3D Radiography**	**Radiographic analysis**<br>- All control teeth healed<br>- Root length/ apical area/ root thickness for both groups<br>- Healing periapical lesion: Control > LPRF<br>- Decreased PAI: Control > LPRF
**Yoshpe et al. 2021 (8)**	REP immature incisor teeth Pulp necrosis + Apical Periodontitis	24 months 30 months	PRF + MTA	**2D Radiography Clinical tests**	**Clinical tests**<br>- No sensitivity to percussion or palpation<br>**Radiographic examination**<br>- No radiolucency,<br>- Apices of both roots were closed with a thicker dentin wall.<br>- Resolution of the periapical lesion<br>- Apical closure
**Jiang et al. 2020 (93)**	REP Immature premolar and incisors teeth after trauma	3,7,11 months 3, 29,44;60 months	Collagen matrix + BC + MTA	**Clinical tests Radiographic (CBCT) examinations**	**Clinical tests**<br>- Electrical pulp testing: 50%<br>- Subjective symptoms: 100% asymptomatic<br>**Radiographic examination**<br>- Healing of the apical lesion: 100%<br>- Detached apical root tip: 100%<br>- Thickening of the root canal wall (main root): 100%<br>- Significantly continued development (main root): 100%<br>- Thickening of the root canal wall (detached root tip): 100%<br>- Significantly continued development (detached root tip): 100%
**Sabeti et al. 2021 (94)**	REP Immature incisors teeth after trauma	18 months	BC + MTA	**Clinical tests Radiographic (CBCT) examinations**	**Clinical tests**<br>- Sensibility testing / electric pulp testing: positive<br>- Asymptomatic

					**<u>Radiographic examination</u>**<br>- No symptoms
**Yoshpe et al. 2020 (117)**	REP<br>Four cases (5 teeth) of posttraumatic immature incisors teeth diagnosed with necrotic pulp and apical Periodontitis or chronic apical abscess	36 months	PRF + Resorbable Collagen Plug + Biodentine	**Clinical tests 2D Radiography**	**<u>Clinical tests</u>**<br>- asymptomatic and tested negatively on thermal testing with percussion and palpation responses<br><br>**<u>Radiographic examination</u>**<br>- Calcified bridge 3 mm coronal to the apex<br>- Resorption appeared to be arrested<br>- Normal periodontal ligament surrounded the mesial and apical aspects of the root<br>- Intact periodontal ligament surrounding the root<br>- Resorption had been arrested<br>- Calcified substance filled the root canal; in the middle third of the root canal, a calcified bridge had developed
**Cymerman et al. 2020 (120)**	REP<br>1 immature tooth with apical periodontitis<br>3 mature teeth with chronic apical abscess/ apical abscess/ acute apical abscess/ asymptomatic apical	20-72 months	BC + SynOss Putty	**Clinical tests Radiographic examinations CBCT**	**<u>Clinical tests</u>**<br>- No pain on percussion/palpation/biting.<br>- The probing depths and mobility of all teeth were within normal limits (ie, ≤3-mm probing depths and ≤1-mm mobility)<br>**<u>Radiographic examination</u>**<br>- The apical diameters ranged from 1–3.7 mm<br>- All teeth showed complete osseous healing of the periapical lesions<br>- The root canal spaces showed partial to complete mineralization<br>- Varying degrees of apical closure were detected in recall radiographs and CBCT images

	periodontitis				
**Gaviño et al. 2017 (95)**	REP<br>Three mature incisors teeth with apical periodontitis	1, 2, 6, 12, 24, 36, and 50 months	BC + PRP + MTA	**Clinical tests<br>Radiographic examination**	**<u>Clinical tests</u>**<br>- Asymptomatic and exhibited negative percussion, palpation, and thermal tests<br>**<u>Radiographic examination</u>**<br>- Complete bone healing around the root apex and invagination of the bone inside the canal forming irregular calcified structures; a slight increase of root length<br>- Presence of cortical plates and the resolution of the periapical lesion<br>- Repair of the periodontal ligament around the apex<br>- Presence of a calcified structure just below the MTA barrier and irregular calcified tissue over the entire internal root walls in the apical third of the canal<br>- Resolution of the periapical lesion

**Terauchi et al 2021 (96)**	REP<br>Three cases mature molars teeth with apical periodontitis	1 week, 3 months, 24 months	BC + MTA	**Clinical tests**<br>**Radiographic examination**	**<u>Clinical tests</u>**<br>- Asymptomatic and responded to cold, heat, and electric pulp tests<br>**<u>Radiographic examination</u>**<br>- Combined Root Canal Therapies in Multirooted Teeth with Pulpal Disease<br>- Asymptomatic<br>- Improvement of the periradicular alveolar bone and evidence of palatal canal apical narrowing and remineralization with re-establishment of the cortical plate<br>- Presented with minimal (1+) mobility<br>and all periodontal probings were within normal limits<br>- Absence of periapical lesions<br>- Restitution of supporting bone and absence of periapical lesions
**Nazzal et al. 2018 (130)**	REP<br>Traumatized immature teeth with necrotic pulps	24 months	Using Bi-antibiotic past for disinfection Then finish with glass ionomer	**Clinical tests**<br>**2D Radiography**	**<u>Clinical tests</u>**<br>- Cold test : 100% negative<br>- EPT : 45% positive<br>**<u>Radiographic examination</u>**<br>root length / root length:<br>- 1 = 1/4 root length<br>- 2 = 1/2 root length 1%<br>- 3 = 2/3 root length 73%<br>- 4 = complete root length with open apex 26%<br>- Root width Increased 55% / No change 45%<br>- Root length increased 64%/ No change 36%<br>- Apical foramen width Decreased 82% / Increased 18%<br>- Discoloration<br>3 cases failed : lost to follow up/ trauma/ calcific barrier probably due to long-term use of calcium hydroxide

**Jung et al. 2008 (97)**	REP 9 immature premolar teeth with pulpal necrosis and apical periodontitis	12- 60 months	BC + MTA	**2D Radiography**	**Radiographic examination**<br>- Continued development of the apex<br>- Thickening of the dentinal walls were obvious<br>- Dentinal walls displayed continued thickening with closure of the apex<br>- Complete resolution of the radiolucency with continued apical closure
**McTigue et al. 2013 [67]**	REP 32 immature incisors & premolar teeth with pulpal necrosis and apical periodontitis	12- 48 months	BC + MTA	**Clinical tests 2D Radiography**	**Radiographic examination**<br>- Apical healing: 97%<br>- Closure of the root apex : 75%<br>- Root walls thickened : 72%<br>- Root length increased : 66%<br>- Ideal placement level for MTA was approximately 3 mm apical to the CEJ<br>- Calcific bridge formation<br>9 teeth failed to achieve at least 2 of the 3 root maturation parameters: apical closure; root wall thickening; and root lengthening.
**Li et al. 2017 (99)**	REP Immature Permanent premolar Necrotic Evaginated Teeth with or without apical periodontitis	24 months	BC + MTA	**Clinical tests 2D Radiography**	**Clinical tests**<br>- All asymptomatic<br>- 25% responsiveness to the pulp sensibility<br>**Radiographic examination**<br>- 90% No periapical lesion<br>- RRA increased<br>- Apical diameter decreased<br>- Root length Increased

**Saoud et al. 2014 (100)**	REP Immature permanent incisor teeth with necrotic dental pulps.	12 months	BC + MTA	**Clinical tests 2D Radiography**	**Clinical tests**<br>- No signs of tenderness to percussion or palpation, a swelling or sinus tract, or spontaneous pain: 100%<br>- None of the teeth regained responsiveness to pulpal sensibility tests (cold, heat, and electrical) during the one year follow up period. 100%<br>**Radiographic examination**<br>- Complete resolution of periapical lesion<br>- Complete radiographic apical<br>- Apical diameter increased<br>- Length of the root increased<br>- Width of root wall increased<br>- Hard-tissue bridge formation that occurred within the canal and not apex
**Dabbagh et al. 2012 (101)**	REP Immature permanent incisor teeth with necrotic dental pulps.	24 months	BC + MTA	**Clinical tests 2D Radiography**	**Clinical tests**<br>- Cold test<br>- Periodontal probing<br>**Radiographic examination**<br>- Increased root formation<br>**Problems**<br>- Discoloration of the crown;<br>- Failure to produce bleeding<br>- Collapse of the mineral trioxide aggregate (MTA) material into the canal

**Dudeja et al. 2015 (102)**	REP Immature, non-vital permanent incisors teeth	6 months to 36 months	BC + MTA	**Clinical tests 2D Radiography**	**Clinical tests**<br>- Asymptomatic<br>**Radiographic examination**<br>- Increase in root length as well as increase in the thickness of lateral dentinal wall<br>- IOPA showed further increase in root length and thickness of lateral dentinal walls and complete resolution of periradicular radiolucency<br>- Increasing resolution of periradicular radiolucency and almost closure of open apices
**Ulusoy et al. 2017 (103)**	REP Necrotic primary molars	18 months	BC + MTA	**Clinical tests 2D Radiography**	**Clinical tests**<br>- Asymptomatic<br>- No discoloration<br>**Radiographic examination**<br>- Complete resolution of periapical and inter-radicular radiolucency<br>- Evidence of re-established periodontal space along the roots
**Cehreli et al. 2011 (104)**	REP Immature necrotic permanent first incisor and molar molars	10 months	BC + MTA	**Clinical tests 2D Radiography**	**Clinical tests**<br>- Positive to cold test<br>**Radiographic examination**<br>- Increase in the root dimensions<br>- Increase in the root wall thickness and root length<br>- Complete or advanced apical closure.

**Mehrvarzfar et al. 2017 (119)**	REP Necrotic teeth immature	6 months	BC +calcium-enriched mixture (CEM) cement	**Clinical tests 2D Radiography**	**Clinical tests**<br>- Asymptomatic<br>- Not responsive to cold test<br>- Positively to EPT test<br>**Radiographic examination**<br>- Healing of periradicular radiolucency<br>- Thin layer of dentinal bridge formed on CEM plug<br>- Thickening of the lateral dentinal walls<br>- Elongation of the root,<br>- Apical closure with formation of the apical barrier tooth was not responsive to cold test<br>- Positively to EPT test at maximum output power<br>- Second case same as the first but not responsive to EPT and cold tests.
**Sachdeva et al. 2015 (105)**	REP Immature incisor pulp necrosis	3, 6, 12, 24 and 36 months	BC + PRP clot + MTA	**Clinical tests 2D Radiography**	**Clinical tests**<br>- Not responsive to cold tests<br>- Delayed positive on EPT<br>**Radiographic examination**<br>- Resolution of the periapical lesion<br>- Increased thickening of the root walls,<br>- Increased root development<br>- Continued apical closure of the root apex
**Shiehzadeh et al. 2014 (112)**	REP Traumatic injury Pulp necrosis on immature incisor and molar teeth	3, 6, 12, 24, months	BC + DPSCs + (PLGA-PEG) + MTA or GIC	**2D Radiography**	**Radiographic evaluation**<br>- Asymptomatic<br>- No signs of the sinus tract<br>- Indication of continued development of the apex of the tooth around the obturating materials

					- Remained functional with continued bone healing and bone completely healed
**Lin et al. 2014 (106)**	REP<br>Traumatic injury on immature teeth which was avulsed and replanted	24 months	BC + MTA	**Histologic and histobacteriologic observation**	**Histology observation**<br>- Extensive dentin resorption altering the radicular contour<br>- Some areas: calcified tissue resembling cementum covering the resorbed dentin<br>- Fragments of periodontal ligament with no inflammatory cells could be seen attached to this tissue<br>- Root canal was apparently empty, with remnants of an amorphous material (likely MTA)<br>- Apical canal was filled with necrotic debris<br>**Histobacteriology observation**<br>- Bacterial aggregates on the root canal walls<br>- Bacterial staining revealed that the necrotic mass filling the foramen was a thick bacterial biofilm exhibiting different bacterial concentrations<br>- Bacterial cells were predominant over the extracellular matrix in the foraminal area, where the biofilm extended beyond the root canal limits, faced with a concentration of polymorphonuclear leukocytes<br>Failure can be = inadequate root canal disinfection without mechanical debridement

**Becerra et al. 2014 (107)**	REP Immature permanent premolar with chronical apical abscess	12 months, 18 months, 24 months	BC + MTA	**Radiographic observation HE (tooth extraction)**	**<u>Radiographic examination</u>**<br>- Periapical radiolucency decreased.<br>- Asymptomatic<br>**<u>Histologic observation</u>**<br>- Fibrous connective tissue characterized mainly by fibroblasts and collagen fibers<br>- Nonmineralized, with the exception of an island of dystrophic calcification apically, close to the foramen<br>- Apical third of the canal: cementum-like tissue<br>- Tubule-like structures of mineralized tissue or odontoblast-like cells could be observed in the canal.<br>- Mild to moderate concentrations of chronic inflammatory cells: in the apical connective tissue<br>- No odontoblast-like cells (rather only fibroblasts) could be seen layering the newly formed cementum
**Chen et al. 2013 (108)**	REP Necrotic immature permanent premolar tooth	1,3,6,9, 12 months	BC + MTA	**Clinical tests 2D Radiography**	**<u>Clinical tests</u>**<br>- asymptomatic<br>**<u>Radiographic examination</u>**<br>- Resolution of periapical radiolucency<br>- Increased thickening and lengthening of the root showed continual development of the root<br>- Presence of isolated island in the canal and become like a bridge

**Chang et al. 2013 (109)**	REP 3 immature incisor teeth with periapical lesion	36 months	BC + MTA	**Clinical tests CBCT examination**	**<u>Clinical tests</u>**<br>- absence of clinical signs and symptoms with adequate function.<br>**<u>Radiographic examination</u>**<br>- significant reduction of periapical radiolucent lesion was noted and the overfilled MTA was completely surrounded by bony (or bone-like) tissues<br>- Osseous repair and a continuous lamina dura-like structure could be observed along with the extruded MTA.<br>- Complete healing of the periapical lesion with the extruded MTA intermingled with regenerated bone with a periodontal ligament-like space between them
**Lenzi et al. 2012 (110)**	REP 2 immature teeth with crown fracture	21 months	BC + MTA	**Clinical tests 3D Radiography**	**<u>Clinical tests</u>**<br>- Asymptomatic<br>**<u>Radiographic examination</u>**<br>- Distinct thickening of the dentinal walls and closure of the apex of the root + patient remained asymptomatic (1st tooth)<br>- No evidence of revitalization could be seen<br>- Apical radiolucency had resolved completely<br>- Distinct radiopaque barrier was seen at the apical opening, suggesting the formation of a hard-tissue barrier. (2nd tooth failed)
**Shin et al. 2009 (111)**	REP Immature teeth with necrotic pulps	2 - 19 months	BC + MTA	**Clinical tests 2D Radiography**	**<u>Clincal tests</u>**<br>- Asymptomatic<br>- No tenderness to percussion or palpation<br>- Ice test and electric pulp test were negative.<br>**<u>Radiographic examination</u>**<br>- Evidence of complete periradicular bone healing and root maturation<br>- Complete resolution of condensing osteitis

**Bakhtian et al. 2017 (118)**	REP<br>4 immature incisors teeth with necrotic pulps	9 - 18 months	BC + PRF scaffold + Biodentine	**Clinical tests<br>2D Radiography**	**<u>Clinical tests</u>**<br>- Asymptomatic<br>**<u>Radiographic examination</u>**<br>- periapical lesion healed<br>- Apical closure<br>- Root thickening for all cases
**Plascencia et al. 2016 (113)**	REP<br>Crown fracture on immature incisor tooth	20 months	BC + MTA	**Clinical tests<br>2D Radiography<br>Tomographic signs**	**<u>Clinical tests</u>**<br>- No signs and symptoms of endodontic re-infection upon<br>**<u>Radiographic examination</u>**<br>- No signs and symptoms of endodontic re-infection upon
**Shimizu et al. 2013 (127)**	REP<br>Immature incisor tooth with traumatic injury (Pulpal necrosis / chronic apical abscess	12 months / 26-months crown fracture: tooth extraction for histology	BC + light-cured composite restoration (Amelogen Plus)	**2D Radiography<br>Histology of the crown fracture**	**<u>Radiographic examination</u>**<br>- More significant increases in root width<br>- Increase in the radiographic area of the root<br>**<u>Histology observation</u>**<br>- Most apical portion of the canal was filled with newly formed mineralized tissue<br>- Uninflamed vital connective tissue was noted enclosed in the mineralized tissue<br>- Newly formed mineralized tissue appeared to resemble cementum-like tissue with incremental lines and osteocyte-like lacunae<br>- Necrotic tissue was also present between the canal dentin and the mineralized tissue<br>- The apical canal appears obliterated by calcified tissue<br>- No pulp-like tissue characterized by the presence of polarized odontoblast-like cells aligning the dentin-like tissue was observed.<br>- Immunohistochemical staining was only positive for BSP

**Meschi et al. 2016 (136)**	REP<br>Infected<br>Immature permanent molar tooth	11 months	BC + MTA	**2D Radiography<br>Histology<br>Immunohistochemistry**	**Histology observation**<br>HE<br>- Calcified bridge with cell inclusions next to MTA<br>- Connective pulp-like tissue with blood vessels and most apically<br>- Internal resorption of the root dentin and osteodentin apposition (atubular tertiary dentine with cells entrapped)<br>- External root surface resorption and cementum apposition appear underneath the periodontal ligament<br>IHC<br>- CD34+ with the blood vessels<br>- Stromal cells in the PLT reacted positive to the OC staining<br>**Radiographic examination**<br>- Increase root length and in root width
**Chen et al. 2012 (92)**	REP<br>Immature permanent incisor teeth with infected necrotic pulps and apical periodontitis /Abscess	6 to 26 months	BC + MTA	**2D Radiography**	**Radiographic examination**<br>- Increased thickening of the canal walls and continued root maturation<br>- No significant continuation of root development with the root apex<br>- Continued root development with the apical foramen remaining open<br>- Severe calcification (obliteration) of the canal space<br>- Hard tissue barrier formed in the canal between the coronal MTA plug and the root apex.

**Meschi et al. 2018 (72)**	REP<br>Infected immature permanent incisors teeth	3, 6, 12, 24, and 36 months	BC + biodegradable bovine collagen plug + MTA	**Clinical tests / PAI 2D/3D Radiography**	**Clinical tests**<br>- Teeth were asymptomatic<br>- Negatively on carbon dioxide snow and electrical pulp testing.<br>- Discoloration by MTA<br>**Radiographic examination**<br>- Periapical lesion healed<br>- PAI between 1 and 2<br>REP healing<br>- Increased thickening of the root canal walls and continued root maturation<br>- No significant continuation of root development with the root apex becoming blunt and closed<br>- Severe calcification (obliteration) of the root canal space<br>3D analysis by measuring<br>- Increases root hard tissue volume<br>- Increases root length<br>- Increases root area<br>- Decreases Periapical lesion area<br>- Presented bone ingrowth inside the root canal, calcification or no uniform root development<br>fail PAI > 3-5

**Meschi et al. 2019 (125)**	REP<br>Infected immature permanent incisors teeth caused by trauma	3 and 5,5 months<br>6 and 7 months<br>20 & 22 months	BC + biodegradable bovine collagen plug + Portland cement	**Radio 2D/3D + Micro CT<br>Histology<br>Immunohistochemistry**	**<u>Clinical test</u>**<br>- Sensibility test : negative<br>- percussion test : negative<br>- electric test : negative<br>**<u>Radiographic examination</u>**<br><u>CBCT</u><br>- Ankylosed<br>- presence periapical lesion (2nd case)<br>- Periapical lesion healed (3rd case)<br>**<u>Histology observation</u>**<br><u>TCM+ IHC</u><br>- Presence of vessels<br>- Presence of mineralized tissues next to MPC and some mineralized island tissue.<br>- Presence of conjunctive repaired tissue<br><u>Micro-CT</u><br>- presence of calcified bridge MPC<br>Failure due to trauma case 1, No mineralization in the apex case 2, Bacteria invasion case 3.
**Elfrink et al. 2021 (73)**	REP<br>Incisors immature traumatized teeth	6-78 months	BC + MTA	**Photographs<br>2D Radiography**	**<u>Radiographic examination</u>**<br>- Increases Apical closure<br>- Increases root length growth<br>- Increases thickening of the root walls<br>- Decreases apical rarefaction<br>**<u>Photographs observation</u>**<br>- discolored teeth or not3 of 47 failed due to ankyloses

**Pereira et al. 2020 (74)**	REP Traumatized immature incisors teeth	9 to 36 months	BC + collagen fiber + MTA	**Clinical tests 2D Radiography**	**Clinical tests**<br>- 93,75% successful clinical outcomes: asymptomatic<br>**Radiographic examination**<br>- Increase in root length<br>- Increase in root width<br>- Decrease in apical diameter<br>Failure: retraumatized tooth 1/16
**Chrepa et al. 2020 (75)**	REP Immature permanent incisors, premolar and molar teeth with pulp necrosis	12 – 96 months	BC + collaplug MTA vs BC + collaplug + Biodentine	**Clinical tests 2D/3D Radiography**	**Clinical tests**<br>- 43/51 (84,3%) successful outcomes<br>- Reestablishment of pulp sensibility: 54%<br>- Tooth discoloration: 62% (because of MTA)<br>**Radiographic examination**<br>- 91.4% demonstrated an increase in median RRA<br>5 cases showed a median gain in length<br>- Dentin wall thickness increased<br>- Increase in RRA was 20% (9.6%–27.3%)<br>8 fails<br>- use of Ca(OH)2 and a lower concentration of 1.5% NaOCl were associated with a higher risk of failure<br>- long-standing biofilm structure formed as a result of caries and its insufficient disinfection
**Sutam et al. 2018 (133)**	REP Immature teeth with necrotic pulp	/	/	**Clinical tests 2D Radiography**	**Quantitative radiographic measurement methods**<br>- Bose et al's Method: The Percentage Change of the Root Length and Root Width<br>- Alobaid et al's Method (the Percentage Change of the Root Length and Root Width)<br>- Flake et al's Method (RRA Measurement)<br>- In any case of measurement, the % change root length, width and

					radiographic root area increased.<br>- 18.7% failed, as they had to be extracted due to serious root resorption. 3/14 teeth and 1 missed recall
**Mittman et al. 2020 (76)**	REP Traumatized Immature Permanent incisors	22 months	BC + collagen sponge + MTA	**Clinical tests 2D Radiography**	**<u>Clinical tests</u>**<br>- periapical radiolency: improved<br>- alveolar bone loss: 56.3% developed root resorptions<br>- Ankylosis: 31.3% displayed signs of ankylosis<br>- Super/Infraposition<br>- Sensitivity<br>- Percussion<br>- Discoloration: 92.9%<br>**<u>Radiographic examination</u>**<br>- Increased root thickness: no significant<br>- Pulp space : no significant<br>- Dentin wall widths and apical foramen: no significant<br>- Significant difference could only be found in the decrease of apical foramina<br>81.3% of the teeth survived revascularization and regained sensitivity<br>18.7% failed, as they had to be extracted due to serious root resorption.

**Zizka et al. 2021 (98)**	REP Immature permanent teeth with a diagnosis of pulp necrosis with or without periapical radiolucency	3, 6, 9, 12, 18 and 24 months	BC + MTA AAEP protocol vs Modified protocol	**Clinical tests 2D radiography**	**Clinical tests**<br>- signs or symptoms of apical periodontitis<br>- discoloration<br>- PAI<br>Radiographic<br>- root length change<br>- root width change<br>- root area change<br>- most of the cases failed in the first year and mostly in the AAEP (American Association of Endodontists) group where calcium hydroxide and lower concentrations of calcium hydroxide were used<br>- Higher concentration of hypochlorite no adverse effects in the mean of radiographic root length or width, but the change in radiographic root area was noticeable when a low concentration of sodium hypochlorite was used
**Bose et al. 2009 (131)**	REP Immature Teeth with Necrotic Root 40 control cases (20 apexification cases and 20 nonsurgical root canal treatments	0-36 months	TAP vs CaOH2 vs formocresol	**2D analysis with image J**	**Radiographic examination**<br>- root wall thickness: TAP >> Ca(OH)2 or formocresol groups<br>- root canal length : Ca(OH)2 > TAP > Formocresol<br>- Ca(OH)2 was radiographically restricted to the coronal half of the root canal system, it produced better results than when it was placed beyond the coronal half.<br>- Radiographic evidence of hard tissue deposition was noticed

**Linsuwanont et al. 2017 (76)**	REP Immature permanent teeth with a diagnosis of pulp necrosis with or without periapical radiolucency	12-61 months	BC + MTA	**2D/3D Radiography**	**Clinical tests**<br>- asymptomatic<br>**Radiographic examination**<br>- Root length increased<br>- Root width increased<br>- No visible signs of apposition of mineralized tissue<br>- Newly produced mineralized tissue is thickening with slight apposition on root canal wall in apical third.<br>- Mineralization of detached apical papilla<br>76% were considered as successful<br>4 failed cases were diagnosed as chronic apical abscess with the presence of sinus tract<br>1 tooth was associated with a defective restoration
**Estefan et al. 2016 (78)**	REP Immature necrotic permanent maxillary incisors into 2 groups (9-13 years // 14-18 years)	12 months	BC + MTA	**2D radiography**	**Radiographic examination**<br>- An increase in root length: Young > older age groups<br>- An increase in root thickness<br>- A decrease in apical diameter:<br>Failure in 2 of the 35 recalled cases<br>Younger patients are better candidates for REP<br>Diameter: predictability factor 0.5mm successful but e better with diameters > 1mm.
**Peng et al. 2017 (79)**	REP Diagnosis of pulp necrosis on premolar teeth	48 months	BC+ MTA or GIC	**Clinical tests 2D radiography**	**Clinical tests**<br>- Success rate MTA > GIC<br>- discoloration 19 cases for MTA and 4 GIC<br>- Obliteration observed MTA<GIC<br>**Radiographic examination**<br>- Slight increase in root length for MTA and GIC

					- Dentin thickness a little more important for MTA than GIC but not significant.
**Chan et al. 2017 (83)**	REP<br>Immature necrotic permanent molar, premolar and incisors teeth	1, 2, 3, 6, 12, 18, 24, and 30 months	BC + Collaplug + MTA	**Clinical tests<br>2D radiography**	**Clinical tests**<br>- 92,8% cases successful<br>- No positive response<br>**Radiographic examination**<br>- Absence of radiographic pathosis 100%<br>- Significant increases in the average root length<br>- Root thickness area<br>- 30.8% of the cases showing complete apical closure
**Jeeruphan et al. 2012 (81)**	22 calcium hydroxide apexification cases,<br>19 MTA apexification cases, and<br>20 revascularization cases	21-30 months	BC + Collaplug + MTA	**Clinical tests<br>2D radiography**	**Radiographic examination**<br>- Root width was significantly greater REP > Apexification<br>- Increase of root length was significantly greater REP > Apexification<br>- Survival rate of the REP and MTA apexification were greater than apexification calcium hydroxide<br>- Complete healing similar between all groups
**Bukhari et al. 2016 (82)**	REP<br>Necrotic immature premolar and	7-72 months	BC + Bioceramic Putty<br>vs<br>BC + MTA	**Clinical tests<br>2D/3D radiography**	**Clinical and radiographic analysis**<br>- (75%) healed completely<br>- 3 cases (10.7%) failed during the observation period

	molar teeth with or no periapical periodontitis				- 3 cases failed 10% incomplete healing.<br>No specific info
**Chen et al. 2016 [80]**	Immature incisors teeth (21 apexification and 17 REP)	3 to 12 months	MTA apexification vs REP with MTA + BC	**Clinical tests 2D radiography**	**<u>PAI scoring for REP vs Apexification</u>**<br>- Complication for apexification lead to fracture 14%<br>- 17% present obliteration for REP procedure<br>- More cases healed with REP, more cases are diseased with apexification.
**Song et al. 2017 (84)**	REP Necrotic premolar teeth immature permanent and chronical abscess vs apexification	24.9 months	BC + MTA	**Clinical tests 2D/3D radiography**	**<u>Clinical and radiographic examinations</u>**<br>- resolution of apical periodontitis<br>- root development<br>- intracanal calcification<br>73% resolution of apical periodontitis<br>Calcification intracanal: 62% with 28% classified as calcific barrier (severe calcification)<br>72% present canal obliteration
**Shah et al. 2012 (137)**	REP Mature permanent teeth with pulp and Periapical infection	6 – 24 months	SealBio	**2D/3D radiography**	**<u>Radiographic examination</u>**<br>- decreased lesion size<br>- increased HU cementum<br>- increased HU bone<br>- CBCT - PAI (increased density of bone and cementum)
